# Microglial activating transcription factor 3 upregulation: An indirect target to attenuate inflammation in the nervous system

**DOI:** 10.3389/fnmol.2023.1150296

**Published:** 2023-03-23

**Authors:** Seth D. Holland, Matt S. Ramer

**Affiliations:** International Collaboration on Repair Discoveries, The University of British Columbia, Vancouver, BC, Canada

**Keywords:** microglia, ATF3, activating transcription factor 3, innate immune system, central nervous system, adaptive stress response

## Abstract

Activating Transcription Factor 3 (ATF3) is upregulated in reaction to several cellular stressors found in a wide range of pathological conditions to coordinate a transcriptional response. ATF3 was first implicated in the transcriptional reaction to axotomy when its massive upregulation was measured in sensory and motor neuron cell bodies following peripheral nerve injury. It has since been shown to be critical for successful axon regeneration in the peripheral nervous system and a promising target to mitigate regenerative failure in the central nervous system. However, much of the research to date has focused on ATF3’s function in neurons, leaving the expression, function, and therapeutic potential of ATF3 in glia largely unexplored. In the immunology literature ATF3 is seen as a master regulator of the innate immune system. Specifically, in macrophages following pathogen or damage associated molecular pattern receptor activation and subsequent cytokine release, ATF3 upregulation abrogates the inflammatory response. Importantly, ATF3 upregulation is not exclusively due to cellular stress exposure but has been achieved by the administration of several small molecules. In the central nervous system, microglia represent the resident macrophage population and are therefore of immediate interest with respect to ATF3 induction. It is our perspective that the potential of inducing ATF3 expression to dampen inflammatory microglial phenotype represents an unexplored therapeutic target and may have synergistic benefits when paired with concomitant neuronal ATF3 upregulation. This would be of particular benefit in pathologies that involve both detrimental inflammation and neuronal damage including spinal cord injury, multiple sclerosis, and stroke.

## 1. Introduction

**Figure 1:**
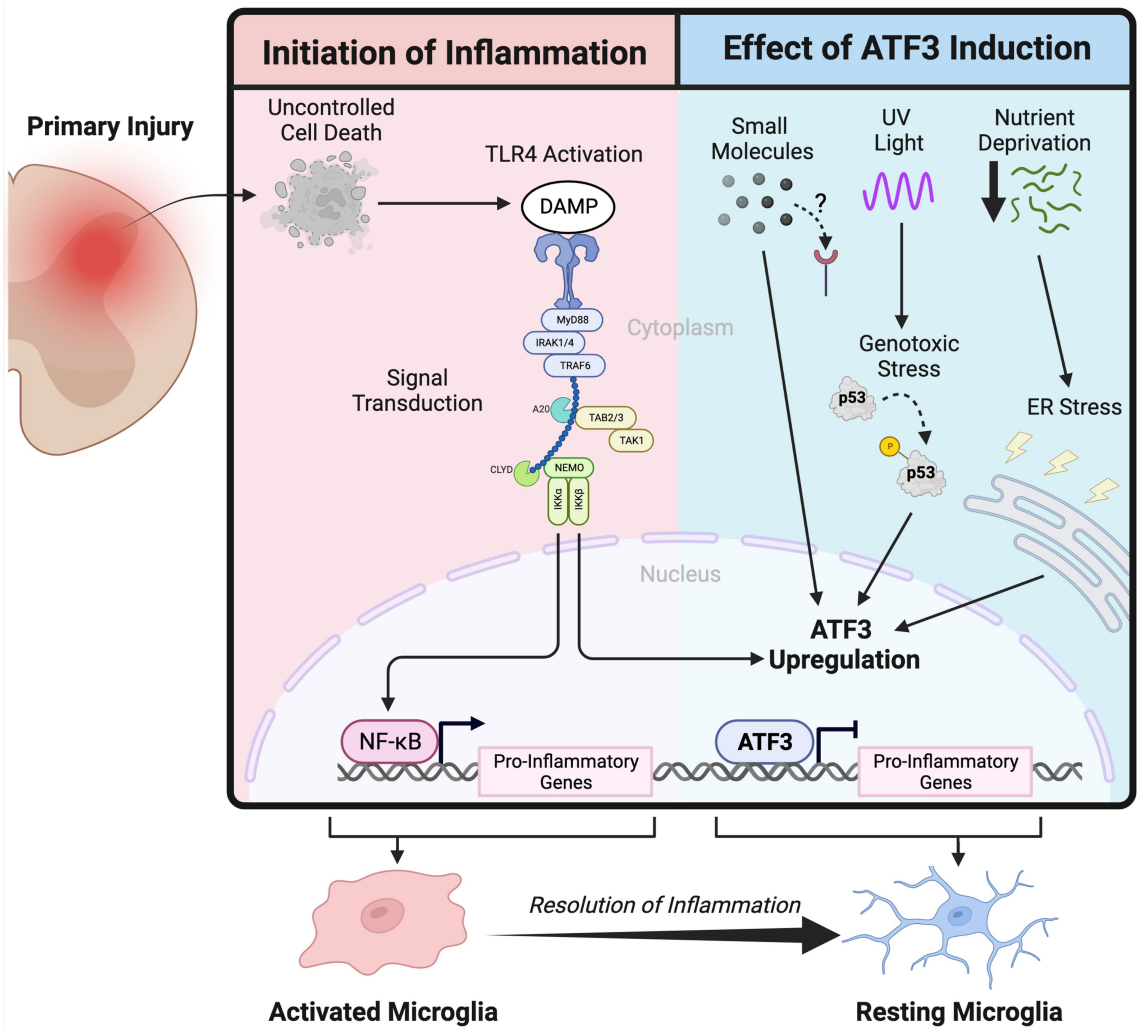
Schematic depicting the activation of the innate immune system following primary injury in the nervous system and the inflammation attenuation effect of Activating Transcription Factor 3 upregulation.

Activating Transcription Factor 3 (ATF3) is a basic leucine zipper (bZIP) transcription factor (Hai et al., 1989) that is immediately upregulated in response to cellular stress (Liang et al., 1996; Hai et al., 1999; Kristensen et al., 2013) to regulate target gene expression. Since ATF3’s precise DNA binding site depends on which bZIP transcription factor it dimerizes with (Tsukada et al., 2011; Rodríguez-Martínez et al., 2017), ATF3’s effect on cellular phenotype is dependent on its context. Given the wide range of stimuli that can trigger a cellular stress response, ATF3 is relevant in a number of pathologies (Allen-Jennings et al., 2002; Wu et al., 2010; Li et al., 2017). ATF3 was first identified in the injured nervous system when its robust upregulation was observed following peripheral nervous system injury in the cell bodies of motor and primary afferent neurons (Tsujino et al., 2000). Since then, it has been shown to contribute to the successful regeneration of axotomized neurons in the peripheral nervous system (PNS) (Gey et al., 2016; Holland et al., 2019); classifying it as a regeneration associated gene. Despite the failure of ATF3 overexpression to initiate a successful neuronal regenerative response in the central nervous system (CNS) (Seijffers et al., 2007), it may still have important prosurvival functions in neurons (Kole et al., 2020; Seijffers et al., 2014). The majority of research done to date on ATF3 and its effect in the nervous system has focused on its neuronal functions, leaving the role of ATF3 in glia largely unexplored (see Figure 1).

## 2. ATF3 as an immune regulator

In the immunologic context, ATF3 is considered a negative regulator of innate immune activation (Gilchrist et al., 2006; Suganami et al., 2009). Macrophages, following damage associated molecular pattern (DAMP) or pathogen associated molecular pattern activation (PAMP) of toll-like receptor 4 (TLR4), initiates a signal transduction cascade that ultimately results in a pro-inflammatory phenotype and the secretion of inflammation propagating signals (Gong et al., 2019; Orecchioni et al., 2019). ATF3 is upregulated in response to TLR4 activation where it functions to suppress pro-inflammatory gene expression, at least partially, through the regulation of histone acetylation (Nguyen et al., 2020). The importance of ATF3’s immunosuppressive function is highlighted in several the disease models where a loss of ATF3 is detrimental including endotoxic shock (Hoetzenecker et al., 2011), atherosclerosis (Gold et al., 2012), and ischemia reperfusion injury (Rao et al., 2014).

ATF3 is also implicated in regulating the innate immune reaction to microbial infection by coordinating the macrophage interferon (IFN) response. ATF3 deficient macrophages following TLR3 or STING stimulation increase IFNβ production compared to wildtype cells (Labzin et al., 2015). IFNβ also increases ATF3 expression (Labzin et al., 2015), suggesting that much like in TLR4 signaling, ATF3 functions as part of an IFN negative feedback loop. Interestingly, using a neuronal cell line that is deficient in IFN I synthesis ATF3 upregulation is observed in response to viral infection (Sood et al., 2017); implying the IFN signaling is not strictly required for inducing ATF3 expression. The demonstration of ATF3 upregulation *via* multiple pattern recognition receptors (TLR4, TLR3, STING) further supports the idea that ATF3 is a hub gene that broadly functions to mitigate an excessive macrophage inflammatory response.

The immunomodulatory function of ATF3 however is not limited to macrophages: ATF3 deficient neutrophils produce excessive CCL2 (a chemokine) but paradoxically are deficient in chemotaxis (Boespflug et al., 2014). Mice with ATF3 deficient natural killer (NK) cells control murine cytomegalovirus better than wildtype NK cells, likely through increased IFNγ production (Rosenberger et al., 2008). In T cells, ATF3 is upregulated upon CD4+ Th1 but not Th2 differentiation; notably a knockdown of ATF3 function in these differentiated cells correspond to decreased IFNγ production (Filén et al., 2010). ATF3 regulates the phenotype of a variety of immune cells however its precise function is dependent on the exact cell type and context.

## 3. Means of ATF3 upregulation

PAMP or DAMP activation is one avenue by which ATF3 can be upregulated, but given its role responding to cellular stress there are additional signals that will induce ATF3. Ultraviolet and ionizing radiation induce ATF3 expression in response to DNA damage through both p53 dependent and independent pathways (Fan et al., 2002). Given its association with DNA damage it is no surprise that ATF3 is overexpressed in cancer (Yan et al., 2017) although its function is dependent on the degree of malignancy (Yin et al., 2008). Amino acid or glucose deprivation, and the latter’s subsequent stress on the endoplasmic reticulum is another pathway by which ATF3 upregulation has been achieved (Pan et al., 2003). The multiple avenues that lead to its upregulation has given rise to the idea that ATF3 is the hub of an adaptive response network that ultimately functions to counteract inflammation (Hai et al., 2010).

In the literature there have been reports of small molecules that induce ATF3 expression with varying results. One of the first uses of an exogenous molecules demonstrated to upregulate ATF3 was anisomycin (Liang et al., 1996), an antibiotic and partial protein synthesis inhibitor, and at low concentrations is used to activate stress response kinases (Kallunki et al., 1994). Utilizing an unbiased screen, the topoisomerase I inhibitor camptothecin was identified as another small molecule capable of upregulating ATF3, and when tested *in vivo* was found to enhance peripheral nerve regeneration (Cheng et al., 2021). Dimethyl itaconate, a cell permeable electrophile was used to upregulate ATF3 and shown to attenuate inflammation in activated macrophages (Bambouskova et al., 2018). These examples prove the principle that ATF3 upregulation is achievable through the administration of small molecules; making ATF3 a potential indirect drug target.

## 4. Aberrant innate immune activation in the CNS

In the CNS microglia are effectively the resident macrophages and represent the primary regulators of the innate immune response (Li and Barres, 2017; Bachiller et al., 2018). While microglial function includes synapse pruning and modulation, they are classically known to survey their environment and initiate an innate immune response if infection or damage is detected. This innate immune reaction can be paradoxically detrimental as it may propagate the initial insult and contribute to a subsequent wave of injury further damaging the parenchyma. An aberrant activation of the innate immune system is a unifying underlying pathology in several diseases. Following a spinal cord injury (SCI), the initial damage to the neural tissue results in uncontrolled cell death that triggers an inflammatory response thought to contribute to the larger secondary wave of injury (Donnelly and Popovich, 2008); mitigating this inflammatory response has been identified as a potential neuroprotective strategy (Kwon et al., 2004). Multiple Sclerosis is an autoimmune disease that targets the myelin sheath resulting in focal inflammatory lesions throughout the CNS (Filippi et al., 2018); several immunomodulatory drugs are currently in use to mitigate the damage and functional impairment exacerbated by the uncontrolled inflammation (Faissner et al., 2019). Much like in SCI, acute ischemic stroke and intracerebral hemorrhage begins as an initial insult that propagates to a secondary injury, a component of which is caused by uncontrolled inflammation (Shi et al., 2019). A strategy to mitigate the microglial inflammatory response would be broadly applicable to a number of CNS diseases by targeting a common pathology.

## 5. Discussion

It is the authors’ perspective that targeting microglial ATF3 upregulation to mitigate inflammation is an unexplored therapeutic avenue across a range of CNS disease. Utilizing small molecules to induce ATF3 in addition to the canonical PAMP/DAMP signaling has been shown to be feasible and blood brain barrier permeability may not be necessary as it is already compromised in many of the applicable conditions. While ATF3 may already be elevated in sterile microglial activation, targeting additional ATF3 upregulation may result in a faster resolution of inflammation, more cells ceasing to release pro-inflammatory cytokines, or providing a different cellular context to alter the precise downstream regulatory effects. Achieving microglial ATF3 induction may also have synergistic benefits when paired with concomitant neuronal ATF3 upregulation given its well-established role as a regeneration associated gene.

## Data availability statement

The original contributions presented in the study are included in the article/supplementary material, further inquiries can be directed to the corresponding authors.

## Author contributions

All authors listed have made a substantial, direct, and intellectual contribution to the work and approved it for publication.

## Funding

SH is supported by a 4 Year Fellowship from the University of British Columbia and by a Doctoral Studentship from the Multiple Sclerosis Society of Canada. MR receives funding for ATF3 research from the Craig H Nielsen Foundation (Grant #647220).

## Conflict of interest

The authors declare that the research was conducted in the absence of any commercial or financial relationships that could be construed as a potential conflict of interest.

## Publisher’s note

All claims expressed in this article are solely those of the authors and do not necessarily represent those of their affiliated organizations, or those of the publisher, the editors and the reviewers. Any product that may be evaluated in this article, or claim that may be made by its manufacturer, is not guaranteed or endorsed by the publisher.

## References

[ref1] Allen-Jennings A. E. Hartman M. G. Kociba G. J. Hai T. (2002). The roles of ATF3 in liver dysfunction and the regulation of phosphoenolpyruvate carboxykinase gene expression. J. Biol. Chem. 277, 20020–20025. doi: 10.1074/jbc.M200727200, PMID: 11916968

[ref2] Bachiller S. Jiménez-Ferrer I. Paulus A. Yang Y. Swanberg M. Deierborg T.. (2018). Microglia in neurological diseases: a road map to brain-disease dependent-inflammatory response. Front. Cell. Neurosci. 12:488. doi: 10.3389/fncel.2018.00488, PMID: 30618635 PMC6305407

[ref3] Bambouskova M. Gorvel L. Lampropoulou V. Sergushichev A. Loginicheva E. Johnson K.. (2018). Electrophilic properties of itaconate and derivatives regulate the IκBζ–ATF3 inflammatory axis. Nature 556, 501–504. doi: 10.1038/s41586-018-0052-z 29670287 PMC6037913

[ref4] Boespflug N. D. Kumar S. McAlees J. W. Phelan J. D. Grimes H. L. Hoebe K.. (2014). ATF3 is a novel regulator of mouse neutrophil migration. Blood 123, 2084–2093. doi: 10.1182/blood-2013-06-510909, PMID: 24470589 PMC3968391

[ref5] Cheng Y. C. Snavely A. Barrett L. B. Zhang X. Herman C. Frost D. J.. (2021). Topoisomerase I inhibition and peripheral nerve injury induce DNA breaks and ATF3-associated axon regeneration in sensory neurons. Cell Rep. 36:109666. doi: 10.1016/j.celrep.2021.109666, PMID: 34496254 PMC8462619

[ref6] Donnelly D. J. Popovich P. G. (2008). Inflammation and its role in neuroprotection, axonal regeneration and functional recovery after spinal cord injury. Exp. Neurol. 209, 378–388. doi: 10.1016/j.expneurol.2007.06.009, PMID: 17662717 PMC2692462

[ref7] Faissner S. Plemel J. R. Gold R. Yong V. W. (2019). Progressive multiple sclerosis: from pathophysiology to therapeutic strategies. Drug discovery 18, 905–922. doi: 10.1038/s41573-019-0035-2, PMID: 31399729

[ref8] Fan F. Jin S. Amundson S. A. Tong T. Fan W. Zhao H.. (2002). ATF3 induction following DNA damage is regulated by distinct signaling pathways and over-expression of ATF3 protein suppresses cells growth. Oncogene 21, 7488–7496. doi: 10.1038/sj.onc.1205896 12386811

[ref9] Filén S. Ylikoski E. Tripathi S. West A. Björkman M. Nyström J.. (2010). Activating transcription factor 3 is a positive regulator of human IFNG gene expression. J. Immunol. 184, 4990–4999. doi: 10.4049/jimmunol.0903106 20304822

[ref10] Filippi M. Bar-Or A. Piehl F. Preziosa P. Solari A. Vukusic S.. (2018). Multiple sclerosis. Nat Rev Dis Prim 4. doi: 10.1038/s41572-018-0041-4 30410033

[ref11] Gey M. Wanner R. Schilling C. Pedro M. T. Sinske D. Knöll B. (2016). Atf3 mutant mice show reduced axon regeneration and impaired regeneration-associated gene induction after peripheral nerve injury. Open Biol. 6:160091. doi: 10.1098/rsob.160091, PMID: 27581653 PMC5008009

[ref12] Gilchrist M. Thorsson V. Li B. Rust A. G. Korb M. Roach J. C.. (2006). Systems biology approaches identify ATF3 as a negative regulator of toll-like receptor 4. Nature 441, 173–178. doi: 10.1038/nature04768, PMID: 16688168

[ref120] Gold E. S. Ramsey S. A. Sartain M. J. Selinummi J. Podolsky I. Rodriguez D. J.. (2012). ATF3 protects against atherosclerosis by suppressing 25-hydroxycholesterol-induced lipid body formation. J. Exp. Med. 209, 807–817. doi: 10.1084/JEM.20111202, PMID: 22473958 PMC3328364

[ref13] Gong T. Liu L. Jiang W. Zhou R. (2019). DAMP-sensing receptors in sterile inflammation and inflammatory diseases. Nat. Rev. Immunol. 20, 95–112. doi: 10.1038/s41577-019-0215-7, PMID: 31558839

[ref14] Hai T. Liu F. Coukos W. J. Green M. R. (1989). Transcription factor ATF cDNA clones: an extensive family of leucine zipper proteins able to selectively form DNA-binding heterodimers. Genes Dev. 3, 2083–2090. doi: 10.1101/gad.3.12b.2083, PMID: 2516827

[ref15] Hai T. Wolfgang C. D. Marsee D. K. Allen A. E. Sivaprasad U. (1999). ATF3 and stress responses. Gene Expr. 7, 321–335. PMID: 10440233 PMC6174666

[ref16] Hai T. Wolford C. C. Chang Y. S. (2010). ATF3, a hub of the cellular adaptive-response network, in the pathogenesis of diseases: is modulation of inflammation a unifying component? Gene Expr. 15, 1–11. doi: 10.3727/105221610X12819686555015, PMID: 21061913 PMC6043823

[ref17] Hoetzenecker W. Echtenacher B. Guenova E. Hoetzenecker K. Woelbing F. Brück J.. (2011). ROS-induced ATF3 causes susceptibility to secondary infections during sepsis-associated immunosuppression. Nat. Med. 18, 128–134. doi: 10.1038/nm.2557 22179317 PMC3555699

[ref200] Holland S. D. Ramer L. M. McMahon S. B. Denk F. Ramer M. S. (2019). An ATF3-CreERT2 knock-In mouse for axotomy-Induced genetic editing: proof of principle. ENeuro, 6. doi: 10.1523/ENEURO.0025-19.2019 PMC6464513 30993183

[ref18] Kallunki T. Su B. Tsigelny I. Sluss H. K. Dérijard B. Moore G.. (1994). JNK2 contains a specificity-determining region responsible for efficient c-Jun binding and phosphorylation. Genes Dev. 8, 2996–3007. doi: 10.1101/gad.8.24.2996, PMID: 8001819

[ref180] Kole C. Brommer B. Nakaya N. Sengupta M. Bonet-Ponce L. Zhao. (2020). Activating transcription factor 3 (ATF3) protects retinal ganglion cells and promotes functional preservation after optic nerve crush. Invest. Ophthalmol. Vis. Sci. 61, PMID: 32084268 10.1167/iovs.61.2.31 PMC7326601

[ref19] Kristensen U. Epanchintsev A. Rauschendorf M. A. Laugel V. Stevnsner T. Bohr V. A.. (2013). Regulatory interplay of Cockayne syndrome B ATPase and stress-response gene ATF3 following genotoxic stress. Proc. Natl. Acad. Sci. U. S. A. 110, E2261–E2270. doi: 10.1073/pnas.1220071110, PMID: 23733932 PMC3690876

[ref20] Kwon B. K. Tetzlaff W. Grauer J. N. Beiner J. Vaccaro A. R. (2004). Pathophysiology and pharmacologic treatment of acute spinal cord injury. Spine J. 4, 451–464. doi: 10.1016/j.spinee.2003.07.007 15246307

[ref21] Labzin L. I. Schmidt S. Masters S. L. Beyer M. Krebs W. Klee K.. (2015). ATF3 is a key regulator of macrophage IFN responses. J. Immunol. 195, 4446–4455. doi: 10.4049/jimmunol.1500204, PMID: 26416280

[ref22] Li Q. Barres B. A. (2017). Microglia and macrophages in brain homeostasis and disease. Nat. Rev. Immunol. 18, 225–242. doi: 10.1038/nri.2017.125 29151590

[ref23] Li Y. Li Z. Zhang C. Li P. Wu Y. Wang C.. (2017). Cardiac fibroblast-specific activating transcription factor 3 protects against heart failure by suppressing MAP2K3-p38 signaling. Circulation 135, 2041–2057. doi: 10.1161/CIRCULATIONAHA.116.024599, PMID: 28249877 PMC5542579

[ref24] Liang G. Wolfgang C. D. Chen B. P. C. Chen T. H. Hai T. (1996). ATF3 Gene: Genomic organization, promoter, and regulation. J. Biol. Chem. 271, 1695–1701. doi: 10.1074/jbc.271.3.1695 8576171

[ref25] Nguyen H. C. B. Adlanmerini M. Hauck A. K. Lazar M. A. (2020). Dichotomous engagement of HDAC3 activity governs inflammatory responses. Nature 584, 286–290. doi: 10.1038/s41586-020-2576-2, PMID: 32760002 PMC7725280

[ref26] Orecchioni M. Ghosheh Y. Pramod A. B. Ley K. (2019). Macrophage polarization: different gene signatures in M1(Lps+) vs. classically and M2(LPS-) vs. alternatively activated macrophages. Front. Immunol. 10:1084. doi: 10.3389/fimmu.2019.01084, PMID: 31178859 PMC6543837

[ref27] Pan Y. X. Chen H. Siu F. Kilberg M. S. (2003). Amino acid deprivation and endoplasmic reticulum stress induce expression of multiple activating transcription Factor-3 mRNA species that, when overexpressed in HepG2 cells, modulate transcription by the human asparagine Synthetase Promoter. J. Biol. Chem. 278, 38402–38412. doi: 10.1074/jbc.M304574200, PMID: 12881527

[ref28] Rao J. Qian X. Li G. Pan X. Zhang C. Zhang F.. (2014). ATF3-mediated NRF2/HO-1 signaling regulates TLR4 innate immune responses in mouse liver ischemia/reperfusion injury. Am J Transplant. 15, 76–87. doi: 10.1111/ajt.12954 25359217

[ref29] Rodríguez-Martínez J. A. Reinke A. W. Bhimsaria D. Keating A. E. Ansari A. Z. (2017). Combinatorial bZIP dimers display complex DNA-binding specificity landscapes. elife 6:e19272. doi: 10.7554/eLife.19272, PMID: 28186491 PMC5349851

[ref30] Rosenberger C. M. Clark A. E. Treuting P. M. Johnson C. D. Aderem A. (2008). ATF3 regulates MCMV infection in mice by modulating IFN-γ expression in natural killer cells. Proc. Natl. Acad. Sci. U. S. A. 105, 2544–2549. doi: 10.1073/pnas.0712182105, PMID: 18268321 PMC2268173

[ref31] Seijffers R. Mills C. D. Woolf C. J. (2007). ATF3 increases the intrinsic growth state of DRG neurons to enhance peripheral nerve regeneration. J. Neurosci. 27, 7911–7920. doi: 10.1523/JNEUROSCI.5313-06.2007, PMID: 17652582 PMC6672733

[ref32] Seijffers R. Zhang J. Matthews J. C. Chen A. Tamrazian E. Babaniyi O.. (2014). ATF3 expression improves motor function in the ALS mouse model by promoting motor neuron survival and retaining muscle innervation. Proc. Natl. Acad. Sci. U. S. A. 111, 1622–1627. doi: 10.1073/pnas.1314826111, PMID: 24474789 PMC3910594

[ref33] Shi K. Tian D. C. Li Z. G. Ducruet A. F. Lawton M. T. Shi F. D. (2019). Global brain inflammation in stroke. Lancet Neurol. 18, 1058–1066. doi: 10.1016/S1474-4422(19)30078-X 31296369

[ref34] Sood V. Sharma K. B. Gupta V. Saha D. Dhapola P. Sharma M.. (2017). ATF3 negatively regulates cellular antiviral signaling and autophagy in the absence of type I interferons. Sci. Rep. 7, 8789–8717. doi: 10.1038/s41598-017-08584-9, PMID: 28821775 PMC5562757

[ref35] Suganami T. Yuan X. Shimoda Y. Uchio-Yamada K. Nakagawa N. Shirakawa I.. (2009). Activating transcription factor 3 constitutes a negative feedback mechanism that attenuates saturated fatty acid/toll-like receptor 4 signaling and macrophage activation in obese adipose tissue. Circ. Res. 105, 25–32. doi: 10.1161/CIRCRESAHA.109.196261, PMID: 19478204

[ref36] Tsujino H. Kondo E. Fukuoka T. Dai Y. Tokunaga A. Miki K.. (2000). Activating transcription factor 3 (ATF3) induction by axotomy in sensory and motoneurons: a novel neuronal marker of nerve injury. Mol. Cell. Neurosci. 15, 170–182. doi: 10.1006/mcne.1999.0814, PMID: 10673325

[ref37] Tsukada J. Yoshida Y. Kominato Y. Auron P. E. (2011). The CCAAT/enhancer (C/EBP) family of basic-leucine zipper (bZIP) transcription factors is a multifaceted highly-regulated system for gene regulation. Cytokine 54, 6–19. doi: 10.1016/j.cyto.2010.12.019, PMID: 21257317

[ref38] Wu X. Nguyen B. C. Dziunycz P. Chang S. Brooks Y. Lefort K.. (2010). Calcineurin and ATF3: opposite roles in squamous skin cancer. Nature 465, 368–372. doi: 10.1038/nature08996, PMID: 20485437 PMC3050632

[ref39] Yan F. Ying L. Li X. Qiao B. Meng Q. Yu L.. (2017). Overexpression of the transcription factor ATF3 with a regulatory molecular signature associates with the pathogenic development of colorectal cancer. Oncotarget 8, 47020–47036. doi: 10.18632/oncotarget.16638, PMID: 28402947 PMC5564541

[ref370] Yin X. DeWille J. W. Hai T. (2008). A potential dichotomous role of ATF3, an adaptive-response gene, in cancer development. Oncogene 27, 2118–2127. doi: 10.1038/sj.onc.1210861, PMID: 17952119

